# The Antimicrobial Cathelicidin CRAMP Augments Platelet Activation during Psoriasis in Mice

**DOI:** 10.3390/biom10091267

**Published:** 2020-09-02

**Authors:** Maryam F. Salamah, Thomas M. Vallance, Xenia Kodji, Divyashree Ravishankar, Harry F. Williams, Susan D. Brain, Sakthivel Vaiyapuri

**Affiliations:** 1 School of Pharmacy, University of Reading, Reading RG6 6UB, UK; maryam.f.salamah@gmail.com (M.F.S.); t.m.vallance@pgr.reading.ac.uk (T.M.V.); divyasri.april86@gmail.com (D.R.); harryfonsecawilliams@gmail.com (H.F.W.); 2 Section of Vascular Biology & Inflammation, School of Cardiovascular Medicine & Sciences, King’s College London, London SE1 9NH, UK; xenia_kodji@sris.a-star.edu.sg (X.K.); sue.brain@kcl.ac.uk (S.D.B.)

**Keywords:** platelets, psoriasis, inflammation, thrombosis, thromboinflammation, mCRAMP, LL37, FPR2/ALX

## Abstract

Platelet-associated complications including thrombosis, thrombocytopenia, and haemorrhage are commonly observed during various inflammatory diseases such as psoriasis. Although several mechanisms that may contribute to the dysfunction of platelets during inflammatory diseases have been reported, knowledge on the primary molecules/mechanisms that underpin platelet-associated complications in such conditions is not fully established. Here, we report the significance of the mouse antimicrobial cathelicidin, mouse cathelicidin-related antimicrobial peptide (mCRAMP) (an orthologue of LL37 in humans), on the modulation of platelet reactivity during psoriasis using Imiquimod-induced psoriasis in mice as an inflammatory disease model for psoriasis vulgaris in humans. The activation of platelets during psoriasis is increased as evidenced by the elevated levels of fibrinogen binding and P-selectin exposure on the surface of platelets, and the level of soluble P-selectin in the plasma of psoriatic mice. The skin and plasma of psoriatic mice displayed increased levels of mCRAMP. Moreover, the plasma of psoriatic mice augmented the activation of platelets obtained from healthy mice. The effect of mCRAMP is partially mediated through formyl peptide receptor 2/3 (*Fpr2/3*, the orthologue to human FPR2/ALX) in platelets as a significant reduction in their activation was observed when FPR2/ALX-selective inhibitors such as WRW_4_ or *Fpr2/3*-deficient mouse platelets were used in these assays. Since the level of antimicrobial cathelicidin is increased in numerous inflammatory diseases such as psoriasis, atherosclerosis, and inflammatory bowel disease, the results of this study point towards a critical role for antimicrobial cathelicidin and FPR2/ALX in the development of platelet-related complications in such diseases.

## 1. Introduction

Psoriasis is a chronic inflammatory skin disease affecting 2–4% of the population worldwide and is characterised by increased proliferation and abnormal differentiation of keratinocytes [1]. Psoriasis vulgaris (also known as plaque-like psoriasis) is the most common phenotype of this disease and is characterised by increased redness, thickening, and scaling of the skin in affected areas, all of which are used to assess the severity of psoriasis in clinical practice [2]. The hyperproliferation of keratinocytes in psoriasis is accompanied by the infiltration of various immune cells and the release of inflammatory mediators. Notably, keratinocytes are known as a rich source for antimicrobial peptides because they express more than 20 different types of these peptides [3]. Among these, LL37 (the only cathelicidin expressed in humans), mouse cathelicidin-related antimicrobial peptide (mCRAMP, an orthologue of LL37 in mice), and psoriasin have been reported to be widely implicated in the development of psoriasis and are highly upregulated in psoriatic lesions [4]. Notably, the concentration of LL37 has been reported to reach up to 300 μM in affected skin [5,6]. Although the cathelicidins exhibit antimicrobial activity against bacteria, viruses, fungi, and parasites, they also modulate inflammatory responses primarily via formyl peptide receptor 2/ALX (FPR2/ALX in humans or *Fpr2/3* in mice) [7,8,9]. Notably, a recent study [10] has reported that histone H2A is able exert its antimicrobial activity in bacteria in conjunction with antimicrobial peptides, LL37 and magainin-2. H2A enters into bacteria such as *Escherichia coli* via the pores made by LL37 and/or magainin-2, and impairs the recovery of membrane and inhibits the bacterial transcription via reorganizing the chromosome.

LL37 is predominantly expressed and stored (as a precursor, human cationic antimicrobial protein 18 (hCAP18)) in various immune cells, including neutrophils and macrophages, and platelets [8,11,12]. From these cells, hCAP18 is secreted and processed into LL37 in response to microbial infection, pro-inflammatory stimuli, and injury in keratinocytes in order to augment microbial clearance and inflammatory responses [4,13]. The changes in the expression of LL37 were implicated in the development of several pathological conditions. For example, the level of LL37 is upregulated in chronic inflammatory diseases including inflammatory bowel disease [14], rheumatoid arthritis [15], psoriasis [16], eczema [17], and atherosclerosis [18,19]. Its downregulation has been implicated in enteric infection [20], acute myeloid leukaemia [21], atopic dermatitis [6], and chronic epithelial ulcers [22]. Although there are differences in peptide sequences, mCRAMP exhibits significant similarities to human LL37, which renders it a useful model to investigate the function and regulation of human cathelicidin [23]. Despite detailed research on the roles of cathelicidins on the modulation of inflammatory responses in various pathological settings, specifically psoriasis [12,24], the effects of cathelicidins on the regulation of platelet reactivity and thrombotic complications were largely unknown until recently [8,25].

Platelets (small circulating blood cells) play indispensable roles in the maintenance of vascular integrity and systemic haemostasis [26]. However, the dysregulation of their functions under pathological conditions may result in thrombotic and/or bleeding complications. Notably, platelets are implicated in the development of numerous pathological conditions such as inflammatory diseases including psoriasis, inflammatory bowel disease, and atherosclerosis, where they have been proposed to contribute to the progression of platelet-associated complications although this could be independent from the development of disease pathology [27,28]. Recently, we reported the effects of LL37 in the augmentation of platelet reactivity and thrombus formation, and in the modulation of haemostasis under physiological conditions [8]. By employing pharmacological inhibitors and platelets obtained from *Fpr2/3*-deficient mice, we demonstrated the functional dependence of LL37 on FPR2/ALX. Moreover, a previous study demonstrated the impact of LL37 in the modulation of thrombosis and inflammatory responses under pathophysiological conditions [25]. In order to determine the significance of LL37/mCRAMP and *Fpr2/3* in the modulation of platelet reactivity and haemostasis during pathological conditions, here, we investigated the levels of mCRAMP and the scale of platelet reactivity during psoriasis using Imiquimod (IMQ)-induced psoriasis in mice as a model for psoriasis vulgaris in humans. This study reveals the clinical significance of mCRAMP in the augmentation of platelet reactivity during psoriasis via *Fpr2/3*.

## 2. Methods

### 2.1. Imiquimod-Induced “Psoriasis-Like” Skin Inflammation

Experiments with mice were conducted according to the UK Home Office Animal Procedures (1986) Act. The project was approved by King’s College Animal Care and Ethics committee. C57/BL6J 6–8 week-old mice (Charles River, UK) were used in a method adapted from Kodji et al., 2019 [29]. A 4 cm^2^ mouse dorsal skin area was shaved and depilated (Veet, Massy, France) prior to daily topical treatment of 75 mg of Aldara™ (Imiquimod, IMQ) cream (Meda Pharma, Bishop’s Stortford, UK) or Vaseline for 4 consecutive days following a previously published protocol [30]. Daily measurements, were taken for body weight, skin thickness, and erythema intensity as well as skin scaling between 9 and 10 a.m.

On day 5, samples were collected from the mice and analysed. The spleen was dissected from the mice and weighed with the mass being normalised to the body weight for statistical analysis. Double fold skin thickness was measured using a micrometer (Farnell, UK) with 0.1 mm accuracy and normalised to day 0 skin thickness. Erythema (redness) and desquamation (scaling) was scored using ‘Psoriasis Area and Severity Index’ (PASI) scoring by treatment-blinded assessors.

### 2.2. Tail Bleeding Assay

Tail bleeding assays were performed as described previously [31,32]. The UK Home Office approved the experimental procedures. In brief, Vaseline-control or IMQ-treated mice were anaesthetised using ketamine (80 mg/kg) and xylazine (5 mg/kg) administered intraperitoneally (i.p.) for 20 min prior to the experiment and placed on a heated mat (37 °C). Then 3 mm of tail tip was removed and immersed in sterile saline prior to monitoring the bleeding time. The time taken for bleeding to cease was recorded. The assay was terminated at 20 min.

### 2.3. Mouse Blood Collection and Platelet Preparation

*Fpr2/3*^−/−^ mice [33] on a C57BL/6 background (obtained from William Harvey Research Institute, London, UK) and wild type C57BL/6 mice from Envigo, UK were used in this study. To collect the blood, mice were euthanised with CO_2_ and the blood was collected via cardiac puncture into a syringe containing 3.2% (*w*/*v*) sodium citrate at a 1:9 ratio. The platelet-rich plasma (PRP) was obtained by centrifuging the whole blood for 8 min at 203 *g* at room temperature. Isolated/washed platelets were obtained by resuspending the remaining blood in modified Tyrode’s-HEPES buffer and repeating centrifugation to collect the remaining PRP. The total PRP collected was centrifuged at 1028 *g* for 5 min and the pellet was resuspended in modified Tyrode’s-HEPES buffer at a density of 2 × 10^8^ cells/mL.

### 2.4. Flow Cytometry-Based Assays

In order to measure the levels of fibrinogen binding and P-selectin exposure on the platelet surface, flow cytometry-based assays were performed. PRP or isolated platelets were incubated with FITC-conjugated fibrinogen antibody (1:50) and PE-Cy^5^-conjugated anti-CD62P (P-selectin) (1:50) antibody in the presence or absence of various concentrations of different platelet agonists. These samples were incubated for 20 min at room temperature, then fixed using 0.2% (*v*/*v*) formyl saline. Fixed samples were analysed using an Accuri C6 flow cytometer (BD Biosciences, Wokingham, UK) by counting 5000 events within the gated population for platelets. Normalisation of the data (median fluorescence intensity) obtained by flow cytometry was performed for easy comparison, independent of the level of percentage positive cells (which may not be 100% in all cases) in each experiment. This enabled the determination of the impact of different treatments to the platelets irrelevant to their level of activation as explained in the relevant sections.

### 2.5. Enzyme-Linked Immunosorbent Assays (ELISA)

To investigate the level of mCRAMP or soluble P-selectin (sP-selectin) in the skin and plasma, a direct ELISA was performed using an mCRAMP- or sP-selectin-selective antibody, respectively. Briefly, a 96-well plate was coated with 50 µL of various concentrations of mCRAMP or sP-selectin (for the standard curve), or the Vaseline- or IMQ-treated samples and incubated at 4 °C overnight. The plate was blocked with 150 µL of assay buffer (0.5% (*w*/*v*) bovine serum albumin in phosphate-buffered saline (PBS)) for 1 h at room temperature. Following washing three times with a wash buffer (0.1% (*v*/*v*) Triton X-100 in PBS), anti-mCRAMP or anti-sP-selectin antibodies were added and the plate was incubated for 4 h at room temperature. Then, plates were washed with wash buffer and incubated for 1 h at room temperature with the secondary antibody (goat anti-mouse horseradish peroxidase-conjugated IgG; Life technologies, UK). Plates were washed three more times and 3,3′,5,5′-tetramethylbenzidine (TMB) substrate was added and allowed to incubate at room temperature. The reaction was stopped by the addition of 100 µL stop solution (0.5 M HCl) and absorbance at 450 nm was measured using an ELISA microplate reader (EMax precision plate reader, Molecular Devices, Wokingham, UK).

### 2.6. Statistical Analysis

Data presented in this study are represented as mean ± SEM. For all experiments involving two or more comparisons, a one-way ANOVA with Bonferroni’s post-hoc test was used but when only two groups were being compared, a parametric two-tailed Student’s *t*-test or a non-parametric Mann-Whitney U test was used depending on whether the data were normally distributed or not (respectively). All statistical analyses were performed using GraphPad Prism 7 software (GraphPad Software Inc., San Diego, CA, USA).

## 3. Results

### 3.1. Characterisation of the IMQ Mouse Model of Psoriasis

**Figure 1:**
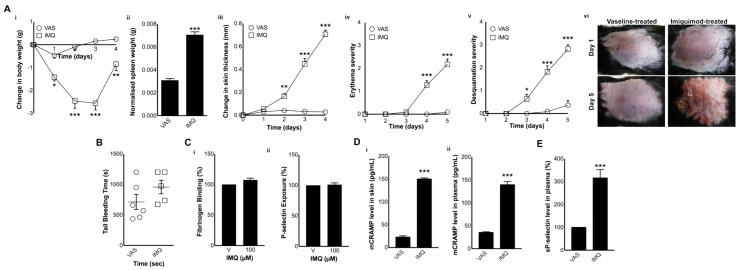
The characterisation, haemostasis, and the expression of mCRAMP in psoriatic mice. (**A**) Characterisation of a psoriasis mouse model was performed by measuring the body weight (Ai), spleen weight (Aii), and skin thickness (Aiii) in Imiquimod (IMQ)-treated mice compared to the controls (Vaseline-treated mice). The ‘Psoriasis Area and Severity Index’ (PASI) scoring was used to rank erythema (Aiv) and desquamation (Av). Representative images display the lesions on the skin of IMQ-treated mice compared to the controls (Avi). Data represent mean ± SEM (*n* = 12 per group). (**B**) The impact of psoriasis in the modulation of haemostasis was analysed in the control or IMQ-treated mice using a tail bleeding assay. Data represent mean ± SEM (*n* = 6 for IMQ-treated, and *n* = 5 for Vaseline-treated mice). (**C**) The inability of 100 µM IMQ (obtained from Meda Pharma, UK) to directly modulate platelet activation was investigated via measuring fibrinogen binding (Ci) and P-selectin exposure (Cii) using undiluted whole blood (incubated at room temperature with occasional shaking) by flow cytometry (by collecting 5000 events within a gated region for platelets). Data represent mean ± SEM (*n* = 4). The median fluorescence intensity (MFI) obtained with vehicle-treated controls was taken as 100% for easier comparison of data from IMQ-treated samples. (**D**) The level of mCRAMP in skin homogenates (Di) or plasma (Dii) samples obtained from IMQ-treated and control mice was analysed using mCRAMP antibodies by an ELISA. Data represent mean ± SEM (*n* = 6 for skin; *n* = 19 for plasma). (**E**) The level of sP-selectin in plasma samples obtained from IMQ-treated and control mice was analysed using sP-selectin antibodies in an ELISA. Data represent mean ± SEM (*n* = 6). *p* values shown are as calculated by one-way ANOVA followed by Bonferroni’s correction in all the experiments except for the data in B and Aii, C, D, and E where a non-parametric Mann–Whitney and a two-tailed unpaired Student’s *t*-test were used, respectively (* *p* < 0.05, ** *p* < 0.001, and *** *p* < 0.0001).

In order to determine the impact of mCRAMP in the modulation of platelet function and haemostasis during psoriasis, we used an animal model with psoriasis-like symptoms. To mimic human plaque-type psoriasis (Psoriasis vulgaris), mice were treated topically with a Vaseline cream containing 5% IMQ, a chemical agent that induces immunomodulation by activating TLR7/8 and adenosine receptors [34,35]. The most commonly used tool to assess the development of psoriasis is the PASI scoring system, which also determines therapeutic efficacy [36]. The PASI scoring system is a five-point scale (0–4) that provides a grade of the average induration (thickness), erythema (redness), and desquamation (scaling) of psoriatic plaques in different body regions including the head, dorsal skin, upper extremities, trunk, and lower extremities [37]. The IMQ-treated mice used in this study displayed all the aforementioned symptoms for psoriasis compared to the control mice (Vaseline-treated). For example, IMQ-treated mice displayed significant differences in the body (Figure 1(Ai)) and spleen (Figure 1(Aii)) weight, and skin thickness (Figure 1(Aiii)) compared to the control mice over 5 days. Moreover, based on the PASI scoring, the clinical manifestations of psoriasis including erythema (Figure 1(Aiv)) and desquamation (Figure 1(Av)) were significantly altered in IMQ-treated mice. Figure 1(Avi) depicts the psoriatic lesions on the dorsal skin of IMQ-treated mice, which are absent in the controls. Together, these data confirm the development of psoriasis-like symptoms in the IMQ-treated dorsal skin of mice, rendering them suitable for further experiments.

### 3.2. Haemostasis Is Not Affected in IMQ-Treated Mice

In order to determine whether the IMQ-treated mice exhibit a direct impact on haemostasis, a tail-bleeding assay was performed. The results demonstrate that IMQ-treated mice did not exhibit a significant difference in the bleeding time compared to the control group (Figure 1B). These data demonstrate that haemostasis is not significantly affected in psoriatic mice.

### 3.3. IMQ Does Not Directly Affect Platelet Activation

The 100 μM IMQ did not induce direct activation of platelets as the level of fibrinogen binding (Figure 1(Ci)) and P-selectin exposure (Figure 1(Cii)) was unaffected when mouse platelets were treated with IMQ. Therefore, the platelet activation observed in psoriatic mice (where IMQ was used for surface treatment) is unlikely to be directly induced by IMQ.

### 3.4. mCRAMP Is Elevated in the Skin and Plasma of IMQ-Treated Mice

Previous studies have reported the overexpression of cathelicidin during psoriasis in humans mainly at local sites of inflammation and lesions [38], and in the circulation [24]. In order to determine whether mCRAMP is overexpressed both locally in the skin and plasma of IMQ-treated mice, the level of mCRAMP was measured in skin homogenates and plasma using immunoassays. The level of mCRAMP was markedly increased in the skin (150 ± 2.4 pg/mL) obtained from IMQ-treated mice compared to the controls (23.1 ± 2.7 pg/mL) (Figure 1(Di)). Moreover, the level of mCRAMP was significantly increased in plasma (139.5 ± 7 pg/mL) obtained from IMQ-treated mice compared to the control (35.4 ± 1.5 pg/mL) (Figure 1(Dii)). These data demonstrate the elevation of mCRAMP levels during the progression of psoriasis both locally in skin lesions and systemically in plasma. Mouse platelets upon activation may also contribute to the increase of mCRAMP in plasma (similar to human platelets [8]) during the progression of psoriasis, however, we were unable to measure this in this study due to limited resources.

### 3.5. The Level of Soluble P-Selectin Is Elevated in Psoriatic Mouse Plasma

The elevated levels of several inflammatory mediators in human plasma during psoriasis were reported previously [27,39]. Some of the notable inflammatory mediators include platelet-derived microparticles, which are also increased in psoriatic patients. The elevated levels of these mediators positively correlated with the PASI scoring and thus disease severity, and their levels were reduced after treatment [27,40,41]. In order to investigate whether the IMQ-treated mice exhibit elevated levels of platelet activation markers in plasma, the level of sP-selectin was measured by ELISA. The plasma obtained from IMQ-treated mice demonstrated significantly increased levels of sP-selectin (at least a 2-fold increase) compared to the control mice (Figure 1E). These data suggest that the elevated level of sP-selectin in circulating plasma during psoriasis may be due to the activation of platelets and also endothelial cells [42].

### 3.6. Platelet Activation Is Increased in IMQ-Treated Mice

**Figure 2:**
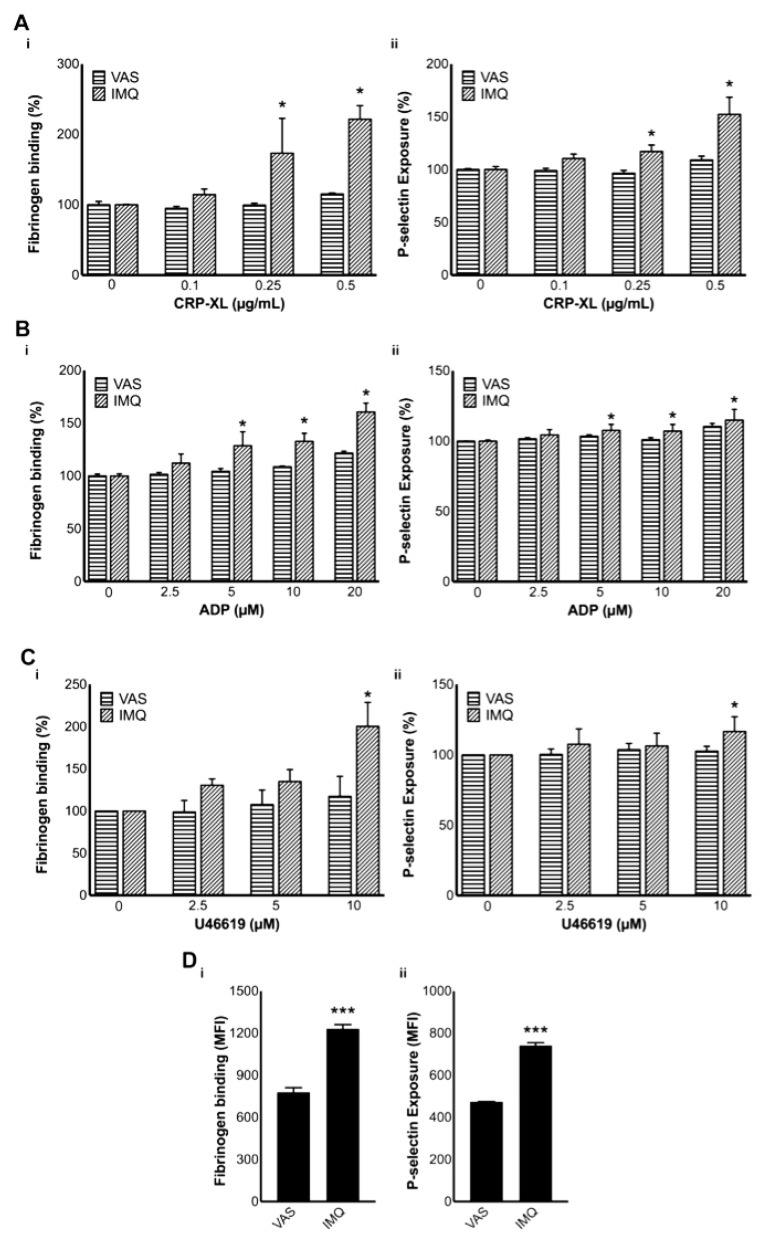
The activation of platelets during psoriasis. The activation of platelets upon stimulation with CRP-XL (*n* = 3) (**A**), ADP (*n* = 4) (**B**), U46619 (*n* = 3) (**C**), or resting platelets (*n* = 4) (**D**) in whole blood obtained from IMQ-treated and control mice was analysed by measuring the level of fibrinogen binding (i) and P-selectin exposure (ii) by flow cytometry (by collecting 5000 events within a gated region for platelets). In (**A**–**C**), the MFI obtained for Vaseline-treated mouse platelets at 0 µM was taken as 100% to normalise the data for easier comparison. *p* values shown are as calculated by two-way ANOVA followed by Bonferroni’s post-hoc test except for the data in (**D**) where a two-tailed unpaired Student’s *t*-test was used (* *p* < 0.05, and *** *p* < 0.0001).

Platelet indices have been demonstrated as useful indicators for the activation of platelets during psoriasis. In addition to the platelet distribution width, the mean platelet volume (MPV) has been reported to be increased in psoriasis [43]. This is indicative of the presence of larger platelets, which are associated with increased reactivity, granule release, and GPIb expression. Moreover, these platelets demonstrate augmented collagen-, ADP-, or thrombin-induced aggregation [44]. In order to confirm whether the platelets in IMQ-treated mice were pre-activated during the progression of the disease, the levels of fibrinogen binding and P-selectin exposure were measured using platelets obtained from these mice compared to the controls by flow cytometry. Furthermore, the platelet activation upon addition of various agonists, such as CRP-XL, ADP, and U46619, was analysed in whole blood obtained from IMQ-treated and control mice. Notably, the levels of fibrinogen binding and P-selectin exposure in platelets obtained from IMQ-treated mice were significantly increased upon treatment with CRP-XL (Figure 2(Ai,Aii)), ADP (Figure 2(Bi,Bii)), and U46619 (Figure 2(Ci,Cii)). Furthermore, the levels of fibrinogen binding (Figure 2(Di)) and P-selectin exposure (Figure 2(Dii)) were increased in resting platelets obtained from IMQ-treated mice. These data corroborate the impact of psoriasis on the modulation of platelet activation, and this may result in thrombosis in microvasculature and other platelet-mediated complications.

### 3.7. Psoriatic Mouse Plasma Activates Healthy Mouse Platelets

**Figure 3:**
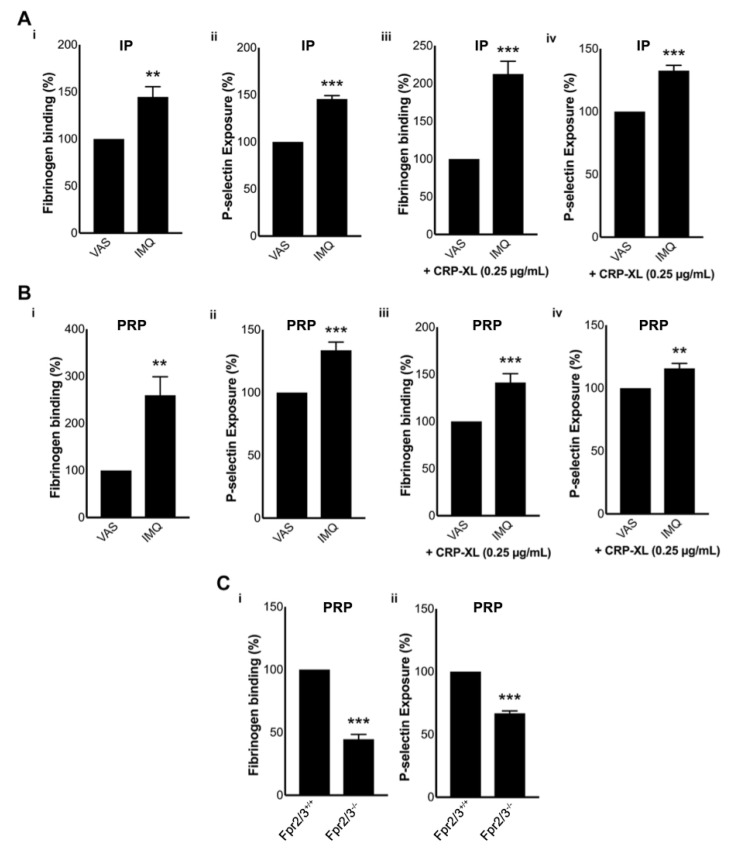
The impact of psoriatic plasma on the activation of healthy mouse platelets. The impact of IMQ-treated mouse plasma on control mouse isolated/washed platelets (IP) was analysed by measuring the levels of fibrinogen binding in the absence (**A**) (Ai) or presence (Aiii) of CRP-XL (0.25 µg/mL) (*n* = 7). Similarly, P-selectin exposure was measured in the absence (Aii) or presence (Aiv) of CRP-XL (0.25µg/mL) (*n* = 7). (**B**) The impact of IMQ-treated plasma on control mouse PRP (in the presence of plasma proteins) was analysed by measuring the levels of fibrinogen binding in the absence (Bi) or presence (Biii) of CRP-XL (0.25 µg/mL) (*n* = 6). P-selectin exposure was also measured in the absence (Bii) or presence (Biv) of CRP-XL (0.25 µg/mL) (*n* = 4). Data represent mean ± SEM. The MFI obtained with Vaseline-treated controls was taken as 100% for easier comparison of data from IMQ-treated samples. (**C**) The impact of IMQ-treated plasma on control and *Fpr2/3*^−/−^ mouse PRP was analysed by measuring the levels of fibrinogen binding (Ci) or P-selectin exposure (Cii) (*n* = 6). The MFI obtained with control mouse platelets was taken as 100% for easier comparison of data from Fpr2/3-deficient mouse platelets. These experiments were performed using flow cytometry by collecting 5000 events within a gated region for platelets. *p* values shown are as calculated by a two-tailed unpaired Student’s *t*-test (** *p* < 0.001, and *** *p* < 0.0001).

Furthermore, we sought to determine whether the plasma of IMQ-treated mice would be able to activate platelets obtained from healthy control mice. Indeed, psoriatic plasma markedly increased the activation of mouse isolated/washed platelets in the absence (Figure 3(Ai,Aii)) or presence (Figure 3(Aiii,Aiv)) of CRP-XL compared to the controls. Similar effects were also observed when healthy mouse PRP (i.e., platelets in the presence of plasma proteins) was used in these assays (Figure 3(Bi–Biv)). These results confirm the ability of psoriatic plasma to induce direct platelet activation in mice. To determine whether these effects were partially/fully mediated by FPR2/ALX, the effects of psoriatic plasma were investigated using *Fpr2/3*^−/−^ mouse platelets. The treatment of psoriatic plasma significantly increased the platelet activation in control mouse platelets, but this effect is largely reduced in platelets obtained from *Fpr2/3*^−/−^ mice (Figure 3(Ci,Cii)). Together, these data demonstrate the involvement of FPR2/ALX in the development of pathogenesis and thrombotic/other platelet-mediated complications during psoriasis. Although we cannot rule out the presence of other molecules and signalling mechanisms in the development of platelet-mediated complications during psoriasis, here we demonstrate the significance of mCRAMP and the involvement (at least partially) of FPR2/ALX in this disease progression.

## 4. Discussion

LL37, a powerful antimicrobial cathelicidin, has been shown to play a significant role in modulating inflammatory responses via leukocytes such as neutrophils and monocytes. Recently, we reported the significance of LL37 in the modulation of platelet reactivity, thrombus formation, and haemostasis [8]. Similar results were also presented by another study to demonstrate the impact of LL37 and mCRAMP on platelet activation and inflammatory responses [25]. Hence, determining the impact of LL37 in the modulation of platelet activation and thrombosis under pathological conditions will reveal novel mechanisms that regulate platelet reactivity under these circumstances. Here, we determined the elevated level of mCRAMP and its activatory role in increasing platelet reactivity during psoriasis using IMQ-induced model in mice. While platelets play indispensable roles in the regulation of haemostasis and immune responses, the dysregulation of their functions results in thrombosis and/or bleeding complications. Thrombosis is considered to be a key factor for several cardiovascular diseases such as myocardial infarction, strokes, and venous thrombosis [45]. However, extensive disseminated intravascular coagulation and/or thrombosis in microvasculature will result in rapid consumption of circulating platelets, which subsequently lead to bleeding complications [28]. Hence, establishing the primary molecules/mechanisms that regulate platelet activation during diverse pathological conditions including psoriasis will aid in the development of improved therapeutic strategies.

Psoriasis is a well-known chronic inflammatory cutaneous disease affecting 2–4% of the worldwide population and is characterised by hyperproliferation and abnormal differentiation of keratinocytes [1]. Psoriatic vulgaris (otherwise known as plaque-like psoriasis) is the most common phenotype of psoriasis and is characterised by increased redness, thickness, and scaling of the skin in affected areas throughout the body, all of which are used to assess the activity and severity of psoriasis in clinical practice [2]. The PASI scoring system provides a grade of the average erythema (redness), induration (thickness), and desquamation (scaling) of psoriatic plaques and this helps to quantify disease progression [36]. Using this scoring system, we confirmed the progression of psoriasis like symptoms in IMQ-induced model in mice under experimental settings. At day 5, all the expected symptoms of psoriasis were developed in comparison to the controls. Notably, the progression of psoriasis did not alter haemostasis in these mice, as the tail bleeding time did not significantly differ in psoriatic mice compared to the controls. The tail bleeding assay reflects the cumulative actions of both coagulation and platelet activation pathways required to maintain haemostasis under physiological settings and, therefore, although platelet activation was observed in all the other assays performed in this study, it did not affect the tail bleeding time in mice.

In order to confirm the functional involvement of circulating platelets in psoriasis, we measured the level of sP-selectin in the plasma, and fibrinogen binding and P-selectin exposure on the surface of platelets obtained from IMQ-treated mice. P-selectin, an adhesion molecule stored in the α-granules of platelets is translocated to their surface and also released into the external milieu upon activation [42]. This, in turn, increases the formation of platelet-leukocyte aggregates and accelerates inflammatory responses [46]. Hence, sP-selectin is considered to be a marker for the activation of platelets in vivo and has been associated with an increased risk of cardiovascular diseases in psoriatic patients [39,47]. In line with this, we determined a significantly increased level of sP-selectin in psoriatic plasma. In addition, at the resting state, psoriatic platelets were found to be significantly activated compared to the control mouse platelets, which may result in thrombus formation in microvasculature. We determined that the platelet activation was not due to a direct effect of IMQ. Moreover, agonist-induced platelet activation was also increased in psoriatic mice. Altogether, these data demonstrate the deleterious effect of psoriasis on the function of circulating platelets.

In addition to the role of inflammatory cells and mediators, antimicrobial peptides have been shown to play an integral role in the pathogenesis of psoriasis [5]. Among the antimicrobial peptides expressed by keratinocytes, cathelicidins (LL37 in humans and mCRAMP in mice) were highlighted as immune modulatory peptides in psoriasis [3,48] and have been shown to be overexpressed with concentrations reaching up to 300 μM in affected skin tissues [6]. In line with the previous studies, here we report the elevated levels of mCRAMP in both psoriatic lesions and plasma of IMQ-treated mice compared to the controls. To determine the impact of elevated level of mCRAMP on platelets, we sought to determine the effect of the psoriatic plasma on healthy mouse platelets. The psoriatic plasma, rich in mCRAMP, induced the activation of mouse platelets and augmented CRP-XL-induced activation of platelets. Hence, the increased level of LL37/mCRAMP under various pathological conditions may able to directly augment platelet activation in order to induce thrombosis and/or subsequent bleeding complications.

LL37 has been reported to mainly mediate its functions via FPR2/ALX in leukocytes and platelets although other receptors were also able to bind this peptide [8,9]. Similar to our previous findings, here we sought to determine whether the effects of mCRAMP are mediated through FPR2/ALX since the level of this peptide in psoriatic mice is markedly increased. Mouse platelets demonstrated reduced activation upon stimulation with psoriatic plasma in *Fpr2/3^−/−^* mice. This suggests the involvement of FPR2/ALX signalling in the LL37/mCRAMP-induced platelet activation during the pathogenesis of psoriasis. Based on our results in this study, we cannot rule out the possibilities of other inflammatory molecules and receptors that may be involved in the activation of platelet function during psoriasis. Further research is required to determine the significance of inflammatory molecules other than LL37 and receptors other than FPR2/ALX in the modulation of platelet activation during psoriasis.

Future studies should focus on investigating the direct impact of mCRAMP on modulating platelet activation during various inflammatory conditions including psoriasis in humans. For example, experiments could be conducted with psoriatic human patients to determine whether their platelet responses are potentiated compared to healthy volunteers and correlate this to bleeding/thrombotic complications observed in those patients. Further research could also include the examination of atherosclerotic lesions in aged mice as mCRAMP is detectable in these lesions and is a known chemotactic agent that can contribute to lesion growth [18,19,49]. Furthermore, the mechanisms (in addition to FPR2/ALX) through which mCRAMP modulate platelet function should be fully established. Further elucidation of the clinical significance of both LL37/mCRAMP and FPR2/ALX in the modulation of platelet activation during inflammatory conditions will enable the better understanding of the pathophysiology of these diseases.

In conclusion, we demonstrate the impact of psoriasis in the modulation of platelet function and this is mainly mediated through increased level of mCRAMP. The markedly increased levels of mCRAMP were observed in psoriatic lesions and plasma of psoriatic mice compared to the controls. Moreover, we demonstrate that the effects of mCRAMP are mediated in part by FPR2/ALX, as the deficiency or blockade of this receptor attenuated the effects of the psoriatic plasma. Haemostasis was not affected in the psoriatic animal model used in this study. The marked level of sP-selectin in psoriatic plasma demonstrates the activation of platelets in psoriatic mice. Psoriatic platelets also demonstrated properties of platelet activation as the levels of fibrinogen binding and P-selectin exposure were increased upon the activation of platelets with CRP-XL, ADP, and U46619 compared to the controls. Moreover, upon the treatment of control platelets with psoriatic plasma, the fibrinogen binding and P-selectin exposure were significantly increased in the presence or absence of CRP-XL. Upon blockade of FPR2/ALX or use of platelets obtained from *Fpr2/3*-deficient mice, the effect of psoriatic plasma on platelet activation was significantly reduced. These confirm the association of cathelicidins and the involvement of FPR2/ALX-mediated signalling in psoriasis. The effect of cathelicidins in the hyperactivity of platelets through FPR2/ALX reveals their significant role in the perpetuation of inflammatory responses in numerous inflammatory diseases where platelets play critical roles. Hence, LL37 and FPR2/ALX may act as potential therapeutic targets to control platelet reactivity and thrombosis during inflammatory diseases such as psoriasis.
